# Vasopressor use as a surrogate for post-intubation hemodynamic instability is associated with in-hospital and 90-day mortality: a retrospective cohort study

**DOI:** 10.1186/s13104-015-1410-7

**Published:** 2015-09-15

**Authors:** Nathan J. Smischney, Onur Demirci, Bryce D. Ricter, Christina C. Hoeft, Lisa M. Johnson, Shejan Ansar, Rahul Kashyap

**Affiliations:** Department of Anesthesiology, Mayo Clinic, 200 First St SW, Rochester, MN 55905 USA; Multidisciplinary Epidemiology and Translational Research in Intensive Care (METRIC), Mayo Clinic, 200 First St SW, Rochester, MN 55905 USA

**Keywords:** Emergent endotracheal intubations, Hemodynamic instability, Mortality, Post-intubation hypotension, Vasopressor use

## Abstract

**Background:**

Evidence is lacking for what defines post-intubation hypotension in the intensive care unit (ICU). If a valid definition could be used, the potential exists to evaluate possible risk factors and thereby improve post-intubation. Thus, our objectives were to arrive at the best surrogate for post-intubation hypotension that accurately predicts both in-hospital and 90-day mortality in a population of ICU patients and to report mortality rates between the exposed and unexposed cohorts.

**Methods:**

We conducted a retrospective cohort study of emergent endotracheal intubations in a medical-surgical ICU from January 1, 2010 to December 31, 2011 to evaluate surrogates for post-intubation hypotension that would predict in-hospital and 90-day mortality followed by an analysis of exposed versus unexposed using our best surrogate. Patients were ≥18 years of age, underwent emergent intubation during their first ICU admission, and did not meet any of the surrogates 60 min pre-intubation.

**Results:**

The six surrogates evaluated 60 min post-intubation were those with any systolic blood pressures ≤90 mmHg, any mean arterial pressures ≤65 mmHg, reduction in median systolic blood pressures of ≥20 %, any vasopressor administration, any non-sinus rhythm and, fluid administration of ≥30 ml/kg. A total of 147 patients were included. Of the six surrogates, only the administration of any vasopressor 60 min post-intubation remained significant for mortality. Twenty-nine patients were then labeled as hemodynamically unstable and compared to the 118 patients labeled as hemodynamically stable. After adjusting for confounders, the hemodynamically unstable group had a significantly higher in-hospital and 90-day mortality [OR (95 % CI); 3.84 (1.31–11.57) (*p* value = 0.01) and 2.37 (1.18–4.61) (p-value = 0.02)].

**Conclusions:**

Emergently intubated patients manifesting hemodynamic instability after but not before intubation, as measured by vasoactive administration 60 min post-intubation, have a higher association with in-hospital and 90-day mortality.

**Electronic supplementary material:**

The online version of this article (doi:10.1186/s13104-015-1410-7) contains supplementary material, which is available to authorized users.

## Background

Airway management using endotracheal intubation is one of the most important skills of the critical care physician. When performed non-emergently in a controlled setting such as the operating room, the complication rate of endotracheal intubation is relatively low; however, the complication rate increases when the procedure is performed outside this controlled environment [1–4]. The incidence of adverse events increases even further when the airway has to be secured emergently [5–8]. The incidence and outcomes of some adverse events related to emergent endotracheal intubation, including immediate airway-related complications, such as hypoxemia, aspiration, airway injury and lost airway, have been described in detail in the literature [1–4, 9]. However, data on the hemodynamic perturbations after the intubation as well as their impact on patients’ outcomes is somewhat limited. Most of the current information on post-intubation hemodynamic instability comes from the emergency department [6, 8, 10, 11]. Studies based in the intensive care unit (ICU) setting found a relationship between post-intubation hemodynamic instability and increased morbidity and mortality; however, these studies failed to establish concrete definitions and validated predictors for mortality or increased length of stay [5, 7, 12].

Perhaps the most important data about post-intubation hemodynamic instability and its impact on patient outcomes comes out of Canada. Green and colleagues performed a structured retrospective chart review on all consecutive adult patients requiring emergent endotracheal intubations over a 16-month period at a tertiary care emergency department [6]. Their consensus definition of hemodynamic instability was a decrease in systolic blood pressure (SBP) to ≤90 mmHg, a decrease 102 in SBP of ≥20 % from baseline, a decrease in mean arterial pressure (MAP) to ≤65 mm Hg, or the initiation of any vasopressor medication at any time in the 30 min following intubation. Out of the 218 patient charts that were reviewed, 96 (44 %) met their criteria of post-intubation hemodynamic instability but despite this high incidence, after controlling for baseline factors in multivariable analysis, the authors couldn’t show an association with increased mortality or hospital length-of-stay. The vasopressors that were used in the study included epinephrine, norepinephrine, phenylephrine, and dopamine, but the authors didn’t compare the differences in outcome when a certain vasopressor was used. Although this study reviewed critically ill patients, the intubation took place in the emergency department and not in the ICU.

Given a possible association between post-intubation hypotension and increased ICU mortality/morbidity, lack of concrete definitions with which to define post-intubation hypotension, and the inherent flaws in the aforementioned studies, more research on this topic is warranted. Thus, we conducted this retrospective cohort study in a population of mixed medical and surgical ICU patients with the primary aim of arriving at the best definition of hemodynamic instability that accurately predicts both in-hospital and 90-day mortality. Our secondary aim was to report in-hospital and 90-day mortality rates between those who became unstable, using our definition, versus those who did not.

## Methods

The study was approved by Mayo Clinic Institutional Review Board for the use of existing medical records of patients who gave prior research authorization (reference number: 12-007113).

### Study design

Retrospective cohort study of critical care patients admitted to a medical and surgical ICU, who underwent emergent intubation, during a 2-year period. This is a retrospective chart review of electronic medical records during the above period.

### Study population

The population under study was obtained retrospectively from two critical care units at Mayo Clinic, Rochester, Minnesota. The two critical care units were a heterogeneous population of medical (65 %) and surgical (35 %) ICU patients admitted during the time period from January 1, 2010 to December 31, 2011. The 2-year time frame was chosen to limit variation in intubation practice as airway techniques (e.g., videolaryngoscopy), as well as resuscitation efforts (e.g., resuscitation protocols) have changed in previous years. The data included only those patients with first-time ICU admissions and excluded 101 patients who did not provide prior research authorization. The total cohort included 6714 consecutive patients admitted to the two intensive care units during the study interval. The cohort was further reduced to 2684 patients who received invasive mechanical ventilation on their first ICU admission during the same period excluding five patients due to age restriction (<18 years of age). Because of evidence suggesting higher mortality with repeated ICU admissions, we only included patients who had emergent endotracheal intubations on their first ICU admission [5, 13]. All emergent intubations analyzed in the current study were performed by trained critical care fellows.

Due to the number of data collection variables we wanted to explore, we utilized two electronic search algorithms for identifying both, if a patient had an emergent endotracheal intubation and when this procedure took place. The electronic search algorithms on the identification and timing of emergent intubations have been published elsewhere and we refer you to these sources [14, 15].

**Fig. 1:**
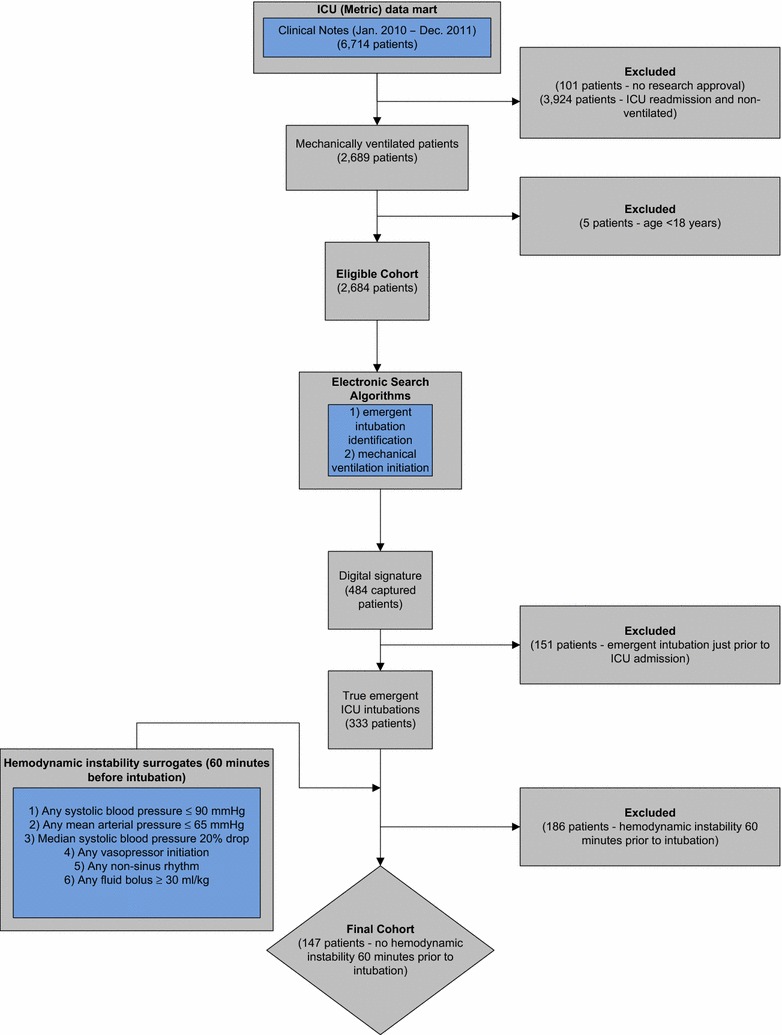
Flow-diagram for the inclusion and exclusion of study patients

Using our previously validated electronic search algorithms, we identified 484 patients as having received emergent endotracheal intubation on their first ICU admission. For data integrity, we manually reviewed all 484 charts. Upon manual review, we identified 333 patients who definitely had received emergent endotracheal intubation on their first ICU admission. Our electronic search algorithm captured an additional 151 charts as having received emergent endotracheal intubation. The additional capture was due to different electronic data systems at our institution where the electronic data pull stopped at one location (e.g., floor) and started to pull data at a different location (e.g., ICU). Therefore, the capture was identified at a time period when the patient was transitioning to the ICU but was endotracheally intubated just prior to ICU arrival because of respiratory distress on the floor. Therefore, our final cohort consisted of 333 patients. Please refer to Fig. 1 for a complete flow diagram of the process.

### Surrogates evaluated for hemodynamic instability exposure

Based on the above literature review, we included six surrogate 171 markers for which we believe correctly identified all critically ill patients developing hemodynamic compromise [5–12]. The six markers included (1) any SBP ≤90 mmHg 60 min post-intubation, (2) any MAP ≤65 mmHg 60 min post-intubation, (3) reduction in median SBP of ≥20 % 60 min post-intubation, (4) any vasopressor administration 60 min post-intubation, (5) any non-sinus rhythm 60 min post-intubation, and (6) fluid administration (crystalloid and/or colloid) of ≥30 ml/kg 60 min post-intubation. In this study, all hemodynamic measurements were recorded from non-invasive devices placed in the upper extremity by the ICU nurse with recordings every 1 min. Vasopressor administration follows no formal protocol at our institution and is at the discretion of the treating clinician.

### Retrospective cohort study with outcome assessment

We choose to focus on two outcome variables, in-hospital and 90-day mortality. We felt that 90-day mortality was an important outcome metric to evaluate in addition to in-hospital mortality as some of the exposures under study (e.g., fluid accumulation) may contribute to increased short-term mortality [16]. Ninety day mortality was measured through the electronic medical record as “alive or deceased”. This was sufficient as all patients in the current study receive care at Mayo Clinic Rochester and we only had 13 subjects lost to follow-up.

Using in-hospital and 90-day mortality as the outcome variable, a univariate model was constructed for each surrogate marker of hemodynamic instability separately. The surrogate marker(s) that were significant at a p-value of ≤0.05 in the univariate model were carried over to the multivariate model.

In the multivariate model, we choose to focus on three possible confounders─age, sepsis as primary diagnosis, and APACHE III score calculated 24 h from ICU admission. We recognize that there are other possible confounders, but we did not want to over-fit our final model given the number of events (death) for in-hospital and 90-day mortality.

We accepted predictors in the final model with a p-value of ≤0.05. The above process of constructing the model with each surrogate marker separately was repeated for the multivariate model, with the addition of the confounders. Using the results from the analysis, we separated the cohort into exposed and unexposed by using surrogate marker(s) of hemodynamic instability that best predicted in-hospital and 90-day mortality.

For the analyses, all information was collected via passive methods with the use of electronic medical records. The data was captured from the Mayo Clinic ICU Data mart. This electronic medical record is reliable and validated. The details of which have been published elsewhere [17]. Four clinicians (BR, CH, LJ, and SA) manually abstracted the electronic medical record of all 333 charts for each of the exposures and were kept blinded to the outcome of interest, mortality. The four clinicians followed a standard operating manual for data collection and were trained in its use through the use of practice charts prior to data collection. Inter-rater agreement (kappa) was not assessed. However, charts flagged by data abstractors who were unsure of data collection were then later confirmed by a third party (O.D.). The outcome was assessed by two critical care physicians (NJS and RK) who were unaware of the exposure status of subjects.

### Statistical analysis

Continuous measurements are expressed as mean ± standard deviation (SD) or median and interquartile (IQR) where appropriate, and were compared for statistical differences using paired t-tests or Mann–Whitney U tests. Categorical variables are reported as counts and percentages and were tested for significance using Chi square or Fisher’s exact tests when applicable. All prognostic surrogates that had a p-value of ≤0.05 determined by the univariate regression model were entered into a multivariate logistic regression model using age, APACHE III score, and sepsis diagnosis as covariates. None of these covariates showed significant collinearity between each other or the surrogate definition used (variance inflation factor <5.0). In-hospital mortality served as the dependent variable and the model was performed by the means of a stepwise backward procedure. Patients who met our best definition of instability were then compared with those who did not meet this definition. Ninety day mortality was then evaluated with Cox proportional hazards using the process above. Thirteen subjects were lost to follow-up. Proportional hazards assumption was evaluated by Schoenfeld residuals for age, APACHE III, sepsis diagnosis and vasopressor use, and there was no evidence suggesting that proportional hazards assumption was violated. Model fit was calculated using Harrell’s c-index [18]. The c-index for the model was 0.74 [95 % CI (0.67–0.80)]. Odds ratios and hazard ratios are presented with 95 % confidence intervals. All reported p-values are two-tailed, and a value ≤0.05 was considered statistically significant. We used SAS version 9.3 and JMP version 9.0 (SAS Institute Inc, Cary, NC, USA) statistical packages for all calculations.

## Results

The final cohort consisted of 147 patients who were in need of emergent endotracheal intubation during their first ICU admission after excluding 186 patients who met any of the aforementioned surrogates. The demographic and clinical characteristics of total study subjects as well as the subsets of stable (unexposed) and unstable (exposed) patients are shown in Additional file 1: Table S1.

For in-hospital mortality, we included all six surrogate markers separately in the univariate model. Significant markers which had an association with in-hospital mortality included initiation of any vasopressor 60 min post-intubation (p-value = 0.01) and any MAP ≤65 mmHg 60 min post-intubation (p-value = 0.02). We then performed a multivariate analysis using three potential confounders of age, sepsis diagnosis, and APACHE III score 24 h from ICU admission with vasopressor and MAP surrogates separately. Initiation of any vasopressor 60 min post-intubation remained significant (p-value = 0.01); however, the marker of any MAP ≤65 mmHg 60 min post-intubation did not (p-value = 0.10).

For 90-day mortality, we repeated the above process for all six surrogate markers in the univariate model using Cox proportional hazards. Significant markers which had an association with 90-day mortality included initiation of any vasopressor 60 min post-intubation (p-value = 0.02), any MAP ≤65 mmHg 60 min post-intubation (p-value = 0.03), and fluid administration of ≥30 ml/kg 60 min post-intubation (p-value = 0.04). We then performed a multivariate analysis using the same three confounders as outlined above for all three surrogates separately. Initiation of any vasopressor 60 min post-intubation remained significant (p-value = 0.02) but fluid administration of ≥30 ml/kg (p-value = 0.11) and any MAP ≤65 mmHg 60 min post-intubation did not (p-value = 0.24). Refer to Additional file 2: Table S2 and Additional file 3: Table S3 for univariate and multivariate model selection.

**Fig. 2:**
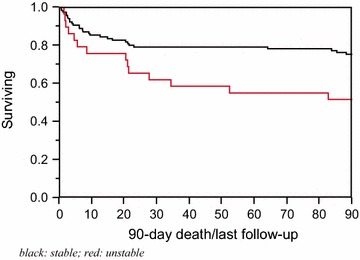
90-day survival curves between those labeled as hemodynamically stable (unexposed) and those labeled as unstable (exposed). Surrogate marker of hemodynamic status: no vasopressor 60 min post-intubation (stable) vs. any vasopressor 60 min post-intubation (unstable)

Based on the above analyses, one surrogate marker was chosen for hemodynamic instability post-intubation that predicted both in-hospital and 90-day mortality; any vasopressor initiation 60 min post-intubation. Of the 147 analyzable patients, 29 met the criteria of any vasopressor 60 min post-intubation. However, only three patients had vasopressor initiation without any documented MAP ≤65 mmHg, and 14 had vasopressor initiation without fluid administration ≥30 ml/kg 60 min post-intubation. Thirty patients had the outcome (death) upon hospital discharge from the time of first ICU admission. Of those 30 patients, 11 had vasopressors started 60 min after intubation as compared to 19 who did not have any vasopressors started 60 min after intubation. Patients found to have any vasopressor administered 60 min post-intubation, using this as a marker for hemodynamic instability, had a significantly higher in-hospital mortality (38 vs. 16 %, p-value = 0.01). After adjusting for age, sepsis diagnosis, and APACHE III score, these patients were more likely to die, OR (95 % CI) = 3.84 (1.31–11.57) (p-value = 0.01) (Additional file 4: Table S4). Using Cox proportional hazards for 90-day mortality, 42 patients had the outcome (death) at 90 days from the time of first ICU admission. Of the 42 patients, 14 patients had any vasopressor initiated 60 min post-intubation versus 28 who did not have any vasopressor 60 min post-intubation. Patients found to have been exposed to this surrogate marker of hemodynamic instability had a significantly higher 90-day mortality (48 vs. 24 %, p-value = 0.02) (Fig. 2). After adjusting for age, sepsis diagnosis, and APACHE III score, these patients were more likely to die, OR (95 % CI) = 2.37 (1.18–4.61) (p-value = 0.02) (Additional file 4: Table S4). Moreover, hospital length-of-stay was significantly longer in those who were exposed to this surrogate marker of hemodynamic instability (Additional file 1: Table S1).

Other potential factors that may be associated with post-intubation hemodynamic instability such as choice of sedative/hypnotic agent administered, pre-existing hypoxemia or hypovolemia and, use of pre-intubation non-invasive ventilation were evaluated and found not to be significantly different between the two groups (Additional file 1: Table S1).

Analyzing the vasopressor group revealed that only 5 of 29 patients required norepinephrine when exposed to a vasopressor 60 min post-intubation as compared to 27 who required phenylephrine. Three of the five patients requiring norepinephrine also required phenylephrine. All 10 deaths came from patients receiving only phenylephrine as the vasopressor of choice. Patients who survived had a median dose of phenylephrine of 300 mcg (187.5–825 mcg) versus 200 mcg (100–1500 mcg) among those who died.

For predicting in-hospital mortality, the model with age, APACHE III score, sepsis diagnosis, and vasopressor exposure 60 min post-intubation (hemodynamic instability) had an area under the curve of 0.80.

## Discussion

We set out to find the best surrogate marker of hemodynamic instability that would accurately predict both in-hospital and 90-day mortality. For both outcomes, only initiation of any vasopressor was significantly associated with in-hospital and 90-day mortality even after adjustment for age, APACHE III score, and sepsis diagnosis. In contrast to studies using different indicators for post-intubation hemodynamic compromise such as SBP ≤90 mmHg and reporting poor outcomes, we did not find significant associations with these commonly reported markers and mortality. Moreover, we included parameters not previously used in the literature and were unable to find associations with these additional factors and mortality.

Our results are consistent with reports of vasoactive exposure and mortality in the critical care environment. For example, Dunser et al. demonstrated that the mean vasopressor load, which included norepinephrine, dopamine, epinephrine, dobutamine, and phenylephrine, was associated with mortality (relative risk 1.83) and adverse events [19]. Furthermore, literature exists for individual vasoactive agents such as dopamine and vasopressin and increased mortality in septic patients and trauma patients [20, 21]. These findings are reported largely in septic patients. However, our study demonstrates the opposite. In the present study, patients that had a favorable outcome included a large proportion of septic patients as compared to the group which had an unfavorable outcome. The finding of very few sepsis patients with increased mortality may represent early recognition and treatment of the disease versus those not labeled with this diagnosis. At our institution, activation alerts for sepsis protocols notify providers early on of potential sepsis patients. These activation alerts are designed to provide care that is consistent with current sepsis guidelines. Therefore, it may be that patients not labeled with this diagnosis upon admission may not receive interventions (e.g. fluid optimization) that would prevent exposure to vasoactive agents for post-intubation hemodynamic instability leading to increased mortality. Even though there was no statistical difference in crystalloid and/or colloid volume during the interval of 24 h pre- and post-intubation between the groups, the hemodynamically stable group (unexposed) had more fluid administration 24 h pre-intubation as compared to the hemodynamically unstable group (exposed) (Additional file 1: Table S1). Although the theory is speculation on the authors’ part, this finding warrants further research to identify the factors that lead to vasoactive exposure post-intubation.

The current study has several limitations. First, the surrogate markers evaluated may not be all inclusive. The markers explored in this study go above and beyond those used in the literature. Therefore, we feel our inclusion criteria were sensitive enough to capture all hemodynamically unstable patients in our ICU. Second, the surrogate markers explored were not mutually exclusive. The reason being is that patients with vasoactive administration are more likely than not to have a MAP ≤65 mmHg at the time of vasoactive administration. This was indeed noted in our population of analyzable study subjects. Therefore, we felt it was not beneficial to have mutually exclusive factors. Third, we cannot exclude the possibility of referral bias in our sample as patients who present to Mayo Clinic may have higher levels of severity than other settings. Fourth, our sample size was limited for some of the above analyses as shown by the wide confidence intervals obtained. In addition, with a larger sample size, we may have seen the other potential explored hemodynamic definitions reach statistical significance. Fifth, our results may not be generalizable to specialized critical care patients such as trauma, pediatrics or neuro critical care patients as these patients were absent from our analysis. Lastly, although we did evaluate potential confounders such as co-morbidities, induction agents, fluid administration, we acknowledge there are other confounders that may have been missed (e.g., correct non-invasive device used, location of measurement, etc.) and thus, the results should be interpreted with caution.

## Conclusions

We have demonstrated that exposure to any vasopressor 60 min post-intubation, as a marker of hemodynamic instability, is associated with increased in-hospital and 90-day mortality in those that are hemodynamically stable prior to intubation. This effect remained despite adjustments in the analyses for potential confounders. The novelty of the present study is the association of vasopressor requirement 60 min post-intubation and 90-day mortality, which to the authors’ knowledge, has not been demonstrated prior. In addition, we have demonstrated that the use of a standard definition for post-intubation hypotension such as SBP <90 mmHg, while may be of value in other settings (i.e., emergence department), was not useful in our analysis. Thus, factors that were relevant in predicting hemodynamic instability surrounding endotracheal intubations in non-ICU settings may not be relevant when analyzing ICU patients. Further research is needed to validate our findings prospectively and to define potential modifiable risk factors that may lead to the requirement of vasoactive medication administration 60 min post-intubation for hemodynamic failure.

## Additional files

**Additional file 1: Table S1.** Sample characteristics grouped by surrogate marker for hemodynamic status post-intubation.
**Additional file 2: Table S2.** Univariate model demonstrating predictive capability of all six surrogate markers of hemodynamic instability for in-hospital and 90-day mortality.
**Additional file 3: Table S3.** Multivariate model demonstrating predictive capability of selected surrogates from univariate model for in-hospital and 90-day mortality after adjusting for age, sepsis diagnosis, and APACHE III score.
**Additional file 4: Table S4.** Comparison of in-hospital and 90-day mortality between those patients labeled as hemodynamically stable (unexposed) and those labeled as hemodynamically unstable (exposed).

## Abbreviations

APACHE: acute physiology and chronic health evaluation
ICU: intensive care unit
MAP: mean arterial pressure
OR: odds ratio
SBP: systolic blood pressure
