# Supplementary material for: Vasopressor use as a surrogate for post-intubation hemodynamic instability is associated with in-hospital and 90-day mortality: a retrospective cohort study
Source: BMC Res Notes. 2015 Sep 15;8:445. doi: 10.1186/s13104-015-1410-7 (PMC4572685; doi:10.1186/s13104-015-1410-7)
Supplement: Supplementary file 1 — Additional file 1: Table S1. Sample characteristics grouped by surrogate marker for hemodynamic status post-intubation. [file 13104_2015_1410_MOESM1_ESM.docx]

Table S1: Sample characteristics grouped by surrogate marker for hemodynamic status post-intubation.^a^

| Variables | Total N=147 | Stable  N=118 | Unstable  N=29 | P-value |
| --- | --- | --- | --- | --- |
| 1. Age (years) | 61.42±15.21 | 60.22±15.96 | 66.28±10.60 | 0.02^b^ |
| 2. Weight (kilograms) | 80.62±22.03 | 81.56±22.46 | 76.79±20.10 | 0.27 |
| 3. Sex (male) | 81 (55) | 68 (58) | 13 (45) | 0.21 |
| 4. SOFA score (day 1) | 7.61±3.42 | 7.53±3.30 | 7.93±3.94 | 0.62 |
| 5. APACHE III score (24 hours from admit) | 81.79±21.79 | 80.59±21.32 | 86.66±23.35 | 0.21 |
| 6. Emergent intubation complication (yes) | 12 (8) | 7 (6) | 5 (17) | 0.05^b^ |
| 7. Airway difficulty (>1 attempt) | 15 (10) | 12 (10) | 3 (10) | 0.98 |
| 8. Reason for intubation |  |  |  |  |
| Airway protection | 74 (50) | 63 (53) | 11 (38) | 0.14 |
| Acute respiratory failure | 114 (78) | 88 (75) | 26 (90) | 0.08 |
| Altered mental status | 26 (18) | 22 (19) | 4 (14) | 0.50 |
| Cardiac arrest | 2 (1) | 1 (1) | 1 (3) | 0.30 |
| 9. Induction agents |  |  |  |  |
| Ketamine | 7 (5) | 4 (3) | 3 (10) | 0.12 |
| Etomidate | 89 (61) | 72 (61) | 17 (59) | 0.81 |
| Propofol | 52 (35) | 40 (34) | 12 (41) | 0.45 |
| Midazolam | 107 (73) | 83 (70) | 24 (83) | 0.18 |
| Fentanyl | 117 (80) | 92 (78) | 25 (86) | 0.32 |
| Paralytic - non-depolarizer | 25 (21) | 18 (15) | 7 (24) | 0.17 |
| Paralytic - depolarizer | 45 (31) | 33 (28) | 12 (41) | 0.16 |
| 10. Sepsis | 45 (31) | 41 (35) | 4 (14) | 0.03^b^ |
| 11. Comorbidities |  |  |  |  |
| Congestive heart failure | 20 (14) | 15 (13) | 5 (17) | 0.52 |
| Ischemic heart disease | 35 (24) | 25 (21) | 10 (34) | 0.13 |
| Chronic obstructive lung disease | 31 (21) | 26 (22) | 5 (17) | 0.57 |
| AKI - emergent intubation | 69 (47) | 57 (48) | 12 (41) | 0.50 |
| Dialysis/CRRT - emergent intubation | 7 (5) | 6 (5) | 1 (3) | 0.71 |
| Pneumonia - emergent intubation | 57 (39) | 45 (38) | 12 (41) | 0.75 |
| End-stage renal disease | 7 (5) | 6 (5) | 1 (3) | 0.71 |
| Cirrhosis | 11 (7) | 11 (9) | 0 (0) | 0.09 |
| Stroke | 12 (8) | 10 (8) | 2 (7) | 0.78 |
| Diabetes mellitus 2 | 29 (20) | 27 (23) | 2 (7) | 0.06 |
| 12. Smoking status |  |  |  |  |
| None | 49 (33) | 43 (36) | 6 (21) | 0.11 |
| Past | 68 (46) | 51 (43) | 17 (59) | 0.14 |
| Current | 29 (20) | 23 (19) | 6 (21) | 0.88 |
| 13. NIV - emergent intubation | 88 (60) | 74 (63) | 14 (48) | 0.19 |
| 14. Total crystalloid volume (ml)^c^ |  |  |  |  |
| Pre-intubation | 1878±2181 | 1900±2187 | 1791±2193 | 0.81 |
| Post-intubation | 3326±2756 | 3129±2678 | 4128±2968 | 0.11 |
| 15. Total colloid volume (ml)^c^ |  |  |  |  |
| Pre-intubation | 160±448 | 172±467 | 110±362 | 0.44 |
| Post-intubation | 252±505 | 256±545 | 234±299 | 0.78 |
| 16. Mechanical ventilation (day) | 4.87±4.81 | 4.54±4.53 | 6.20±5.71 | 0.15 |
| 17. ICU length of stay (day) | 7.40±5.78 | 6.95±5.31 | 9.23±7.22 | 0.12 |
| 18. Hospital length of stay (day) | 17.57±16.03 | 15.76±13.83 | 24.90±21.73 | 0.04^b^ |

*Abbreviations: SOFA: sequential organ failure assessment; APACE: acute physiology and chronic health evaluation; NIV: non-invasive ventilation; AKI: acute kidney injury; CRRT: continuous renal replacement therapy; ml: milliliters*

*^a^ surrogate marker of hemodynamic status: no vasopressor 60 minutes post-intubation (stable) vs. any vasopressor 60 minutes post-intubation (unstable)*

*^b^ indicates significance at p-value of ≤ 0.05*

*^c^ indicates total volume 24 hours pre- and post-intubation*
