# Supplementary material for: Vasopressor use as a surrogate for post-intubation hemodynamic instability is associated with in-hospital and 90-day mortality: a retrospective cohort study
Source: BMC Res Notes. 2015 Sep 15;8:445. doi: 10.1186/s13104-015-1410-7 (PMC4572685; doi:10.1186/s13104-015-1410-7)
Supplement: Supplementary file 2 — Additional file 2: Table S2. Univariate model demonstrating predictive capability of all six surrogate markers of hemodynamic instability for in-hospital and 90-day mortality. [file 13104_2015_1410_MOESM2_ESM.docx]

Table S2: Univariate model demonstrating predictive capability of all six surrogate markers of hemodynamic instability for in-hospital and 90-day mortality.

| In-Hospital Mortality | P-value | OR | 95%CI |
| --- | --- | --- | --- |
| 1. SBP ≤ 90 mmHg | 0.21 | 1.68 | (0.74-3.85) |
| 2. MAP ≤ 65 mmHg | 0.02^a^ | 2.71 | (1.18-6.56) |
| 3. SBP change ≥ 20% | 0.80 | 1.12 | (0.47-2.57) |
| 4. Fluid bolus ≥ 30 ml/kg | 0.09 | 2.00 | (0.88-4.54) |
| 5. Vasopressor initiation | 0.01^a^ | 3.18 | (1.28-7.79) |
| 6. Non-sinus rhythm | 0.09 | 4.22 | (0.75-23.93) |

| 90-Day Mortality | P-value | HR | 95%CI |
| --- | --- | --- | --- |
| 1. SBP ≤ 90 mmHg | 0.07 | 1.79 | (0.96-3.37) |
| 2. MAP ≤ 65 mmHg | 0.03^a^ | 2.01 | (1.07-3.90) |
| 3. SBP change ≥ 20% | 0.40 | 1.32 | (0.69-2.50) |
| 4. Fluid bolus ≥ 30 ml/kg | 0.04^a^ | 1.90 | (1.03-3.50) |
| 5. Vasopressor initiation | 0.02^a^ | 2.24 | (1.15-4.19) |
| 6. Non-sinus rhythm | 0.11 | 2.60 | (0.78-6.48) |

*Abbreviations: HR: hazard ratio; OR: odds ratio; CI: confidence interval*

*^a^ indicates significance at p-value of ≤ 0.05*
