# Supplementary material for: Vasopressor use as a surrogate for post-intubation hemodynamic instability is associated with in-hospital and 90-day mortality: a retrospective cohort study
Source: BMC Res Notes. 2015 Sep 15;8:445. doi: 10.1186/s13104-015-1410-7 (PMC4572685; doi:10.1186/s13104-015-1410-7)
Supplement: Supplementary file 3 — Additional file 3: Table S3. Multivariate model demonstrating predictive capability of selected surrogates from univariate model for in-hospital and 90-day mortality after adjusting for age, sepsis diagnosis, and APACHE III score. [file 13104_2015_1410_MOESM3_ESM.docx]

Table S3: Multivariate model demonstrating predictive capability of selected surrogates from univariate model for in-hospital and 90-day mortality after adjusting for age, sepsis diagnosis, and APACHE III score.

| In-Hospital Mortality | P-value | OR | 95%CI |
| --- | --- | --- | --- |
| 1. MAP ≤ 65 mmHg | 0.10 | 2.16 | (0.87-5.63) |
| 2. Vasopressor initiation | 0.01^a^ | 3.84 | (1.31-11.57) |

| 90-Day Mortality | P-value | HR | 95%CI |
| --- | --- | --- | --- |
| 1. MAP ≤ 65 mmHg | 0.24 | 1.47 | (0.77-2.89) |
| 2. Vasopressor initiation | 0.02^a^ | 2.37 | (1.18-4.61) |
| 3. Fluid bolus ≥ 30 ml/kg | 0.11 | 1.66 | (0.88-3.10) |

*Abbreviations: HR: hazard ratio; OR: odds ratio; CI: confidence interval*

*^a^ indicates significance at p-value of ≤ 0.05*
