# Supplementary material for: Vasopressor use as a surrogate for post-intubation hemodynamic instability is associated with in-hospital and 90-day mortality: a retrospective cohort study
Source: BMC Res Notes. 2015 Sep 15;8:445. doi: 10.1186/s13104-015-1410-7 (PMC4572685; doi:10.1186/s13104-015-1410-7)
Supplement: Supplementary file 4 — Additional file 4: Table S4. Comparison of in-hospital and 90-day mortality between those patients labeled as hemodynamically stable (unexposed) and those labeled as hemodynamically unstable (exposed). [file 13104_2015_1410_MOESM4_ESM.docx]

Table S4: Comparison of in-hospital and 90-day mortality between those patients labeled as hemodynamically stable (unexposed) and those labeled as hemodynamically unstable (exposed).^a^

| In-Hospital Mortality | Stable | Unstable | P-value | OR | 95%CI |
| --- | --- | --- | --- | --- | --- |
| 1. Event (death) | 19 (16) | 11 (38) |  |  |  |
| 2. Non-event (death) | 99 | 18 |  |  |  |
| 3. Total | 118 | 29 |  |  |  |
| 4. Unadjusted analysis |  |  | 0.01^b^ | 3.18 | (1.28-7.79) |
| 5. Adjusted analysis |  |  | 0.01^b^ | 3.84 | (1.31-11.57) |

| 90-Day Mortality | Stable | Unstable | P-value | HR | 95%CI |
| --- | --- | --- | --- | --- | --- |
| 1. Event (death) | 28 (24) | 14 (48) |  |  |  |
| 2. Non-event (death) | 90 | 15 |  |  |  |
| 3. Total | 118 | 29 |  |  |  |
| 4. Unadjusted analysis |  |  | 0.02^b^ | 2.24 | (1.15-4.19) |
| 5. adjusted analysis |  |  | 0.02^b^ | 2.37 | (1.18-4.61) |

*Abbreviations: HR: hazard ratio; OR: odds ratio; CI: confidence interval*

*^a^ surrogate marker of hemodynamic status: no vasopressor 60 minutes post-intubation (stable) vs. any vasopressor 60 minutes post-intubation (unstable)*

*^b^ indicates significance at p-value of ≤ 0.05*
